# Cytokine secretion and NK cell activity in human ADAM17 deficiency

**DOI:** 10.18632/oncotarget.6629

**Published:** 2015-12-16

**Authors:** Pinchas Tsukerman, Eli M. Eisenstein, Maor Chavkin, Dominik Schmiedel, Eitan Wong, Marion Werner, Barak Yaacov, Diana Averbuch, Vered Molho-Pessach, Polina Stepensky, Noa Kaynan, Yotam Bar-On, Einat Seidel, Rachel Yamin, Irit Sagi, Orly Elpeleg, Ofer Mandelboim

**Affiliations:** ^1^ Lautenberg Center for General and Tumor Immunology, The Hebrew University, The BioMedical Research Institute, Hadassah Medical School, Jerusalem, Israel; ^2^ Department of Pediatrics, Hadassah-Hebrew University Medical Center, Mount Scopus, Jerusalem il, Israel; ^3^ Department of Biological Regulation, Weizmann Institute of Science, Rehovot, Israel; ^4^ Monique and Jacques Roboh Department of Genetic Research, Hadassah, Hebrew University Medical Center, Jerusalem, Israel; ^5^ Pediatric Infectious Diseases Unit, Hadassah-Hebrew University Medical Center, Ein Kerem, Kiryat Hadassah, Jerusalem, Israel; ^6^ Department of Dermatology, Hadassah Hebrew University Medical Center, Ein Kerem, Kiryat Hadassah, Jerusalem, Israel; ^7^ Pediatric Hemato-Oncology and Bone Marrow Transplantation Department, Hadassah-Hebrew University Medical Center, Ein Kerem, Kiryat Hadassah, Jerusalem, Israel

**Keywords:** ADAM17 deficiency, NK cells, CD16, ADCC, Immunology and Microbiology Section, Immune response, Immunity

## Abstract

Genetic deficiencies provide insights into gene function in humans. Here we describe a patient with a very rare genetic deficiency of ADAM17. We show that the patient's PBMCs had impaired cytokine secretion in response to LPS stimulation, correlating with the clinical picture of severe bacteremia from which the patient suffered. ADAM17 was shown to cleave CD16, a major NK killer receptor. Functional analysis of patient's NK cells demonstrated that his NK cells express normal levels of activating receptors and maintain high surface levels of CD16 following mAb stimulation. Activation of individual NK cell receptors showed that the patient's NK cells are more potent when activated directly by CD16, albeit no difference was observed in Antibody Depedent Cytotoxicity (ADCC) assays. Our data suggest that ADAM17 inhibitors currently considered for clinical use to boost CD16 activity should be cautiously applied, as they might have severe side effects resulting from impaired cytokine secretion.

## INTRODUCTION

NK cells are innate lymphocytes important in host defense against infections and in immune surveillance of cancerous cells [1, 2]. NK cells kill abnormal cells by using activating and inhibitory receptors [3, 4]. Inhibitory receptors recognize mainly, but not only, MHC class I proteins that are expressed on healthy cells to protect these cells from NK cell attack [5]. The NK activating receptors, which include NKG2D, NKp46, NKp44, NKp30, NKp80, DNAM1, 2B4, and CD16 recognize pathogen-derived ligands, stress-induced molecules, tumor ligands and self-ligands [1]. In activated NK cells, almost all of the above-mentioned NK activating receptors are able to trigger NK cell killing on their own [1, 5, 6]. In contrast, to elicit naïve NK-cell cytotoxicity, triggering of at least two activating receptors is required [7]. The only exception to this, is CD16 (FCIIIγRA), the triggering of which is sufficient on its own in inducing naïve NK cell killing [8]. CD16A, the isoform expressed on NK cells, has two extracellular Ig domains, a short cytoplasmic tail and a transmembrane domain that enables its association with adaptor proteins [9, 10].

ADAM17 is a metallopeptidase, with many known substrates including cytokines and soluble receptors [11] [12]. It was shown that ADAM17 is also capable of cleaving CD16 from the surface of NK cells following activation [13]. Thus, ADAM17 is considered as a negative regulator of CD16 activity. Knockout of ADAM17 in mice is embryonic-lethal and therefore the function of ADAM17 *in vivo*, in mice, was studied using conditional knockouts [14].

Deficiency of ADAM17 in humans has so far been reported in two siblings only [15]. These patients suffered from a severe inflammatory skin phenotype, greatly increased susceptibility to cutaneous and paronychial infections.

Here we identify a new patient suffering from ADAM17 deficiency. We describe his clinical features and study the cytokines secreted from the patients' PBMCs following LPS stimulation and the function of his NK cells.

## RESULTS

### Clinical manifestation

**Figure 1:**
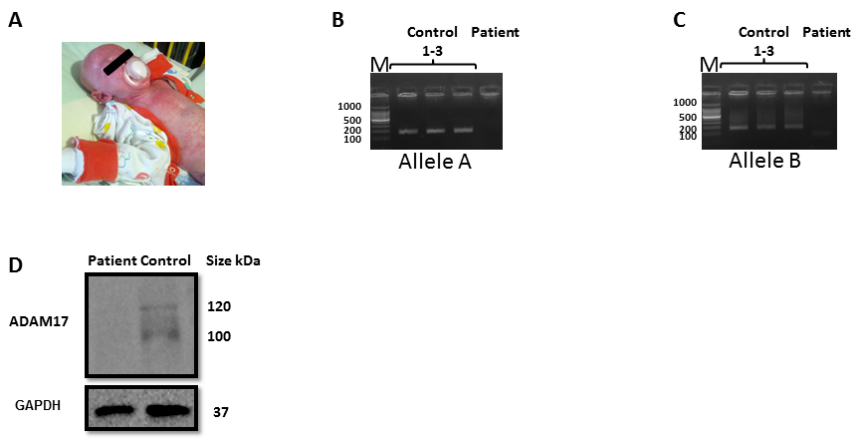
Validation of the ADAM17 deficiency **A.** The patient **B.**-**C.** Genomic region of ADAM17 exon 1, amplified from healthy donors and ADAM17 patient (indicated on the top of the figure). PCR products of Allele A **B.** and Allele B **C.**. The background was adjusted for clarity. **D.** Western blot for the expression of ADAM17 (top) and GAPDH, used as a loading control (bottom) in NK derived from the ADAM17-deficient patient and in NK cells derived from a healthy control. The background was adjusted for clarity.

The patient (Figure 1A) was born at 37 weeks of gestation, weighing 2185 grams to parents who are first cousins. Beginning at one month of age, he suffered from recurrent infections as detailed in the Supplementary Data. The patient died at two years of age of respiratory failure and presumed sepsis.

### Mapping and validation of the ADAM17 deficiency

The susceptibility of the patient to bacterial infections, combined with the acute hyper-inflammation led us to perform an exome-sequencing as previously described [16]. Despite parental consanguinity, he had no rare, homozygous, coding variant which could underlie his disease. However, a single exon, exon 1 of the ADAM17 gene, was totally uncovered in the patient sample, but was well covered (46-48 X) in samples of other patients which were analyzed in parallel. Multiplex Ligation-dependent Probe Amplification (MLPA), using a synthetic probe specific for a unique sequence within exon 1 essentially as in [16], confirmed segregation of the deletion in the family, with an undetectable signal for the patient, a signal of 1.24-1.31 in the DNA samples of the parents and a signal of 1.98-2.17 in ethnically matched control samples.

Specific primers for the deletion region were designed; genomic DNA was extracted from the patient and from three healthy donors, and was amplified using the specific primers. PCR amplification produced a distinct product for the healthy donors allele A, (Figure 1B, lanes 1-3) and allele B, (Figure 1C lanes 1-3), while no amplification product is visible from the patients' DNA. Next, we validated the lack of ADAM17 expression at the protein level. Since we could obtain only few milliliters of blood from the patient we had to grow the immune cells of the patient to obtain sufficient amount of cells. For this, we isolated NK cells from the patient and from two healthy donors and grow them in the presence of IL-2. As can be seen in Figure 1D both the precursor form (around 120 kDa) and the catalytic form (around 100 kDa) of ADAM17 were absent from the patients' NK cells.

### Impaired cytokine secretion in absence of ADAM17

**Figure 2:**
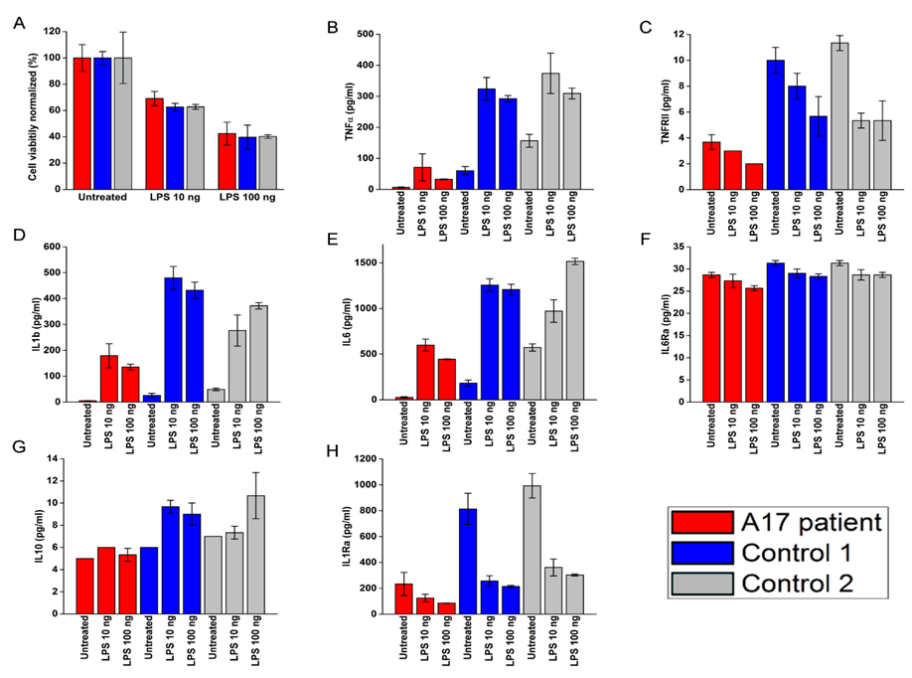
LPS effect on viability and cytokine secretion by the patients PBMCs **A.**-**H.** PBMCs viability **A.** and cytokine secretion **B.**-**H.**. Patients (red bars) and two healthy donors (blue and grey bars). The cytokines are indicated in the Y axis.

ADAM17 is known for its role in modulating the secretion of various cytokines during inflammation [11]. We thus tested total PBMCs derived from the patient and two healthy donors for cell viability and cytokine secretion, with or without prior stimulation by various doses of LPS. The patients PBMCs maintained similar viability compared to both healthy donors PBMCs (Figure 2A). However, (with the exception of IL-6Rα), most of the cytokines and receptors tested (Figure 2B–2H) were secreted in significantly ower amounts by the patient PBMCs (the p-values are found in Supplementary Table 1).

### Phenotypic and functional effect of the deletion on the activity of NK cells

**Figure 3:**
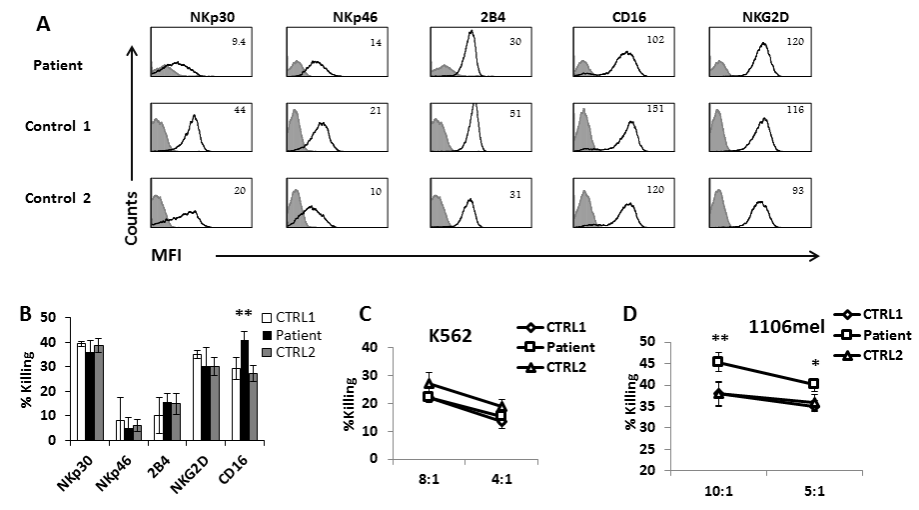
NK cell characterization of ADAM17-deficient patient **A.** FACS analysis of the indicated NK cell receptors (black empty histograms) expressed by IL-2 activated bulk NK cell cultures derived from the ADAM17 patient (top) and from two healthy control donors. The Median Fluorescence Intensity (MFI) is indicated in the figure. The filled grey line histogram is isotype control. Figure shows combined data from 3 independent experiments **B.** Redirected killing assay. IL-2 activated bulk NK cell cultures derived from the ADAM17 patient (black bars) and from two healthy control donors (white and grey bars) were incubated with ^35^S labeled P815 cells at Effector to Target (E:T) ratio of 8:1 in presence of mAbs indicated at the X axis. The killing of P815 cells in presence of mIgG was subtracted from all values. Shown is one representative experiment of three performed. **p < 0.02 by a two tailed student T-TEST. **C.**-**D.** Direct killing assay: IL-2 activated bulk NK cell cultures derived from the ADAM17 patient and from two healthy control donors were incubated with ^35^S labeled K562 cells **C.** or 1106mel cells **D.** at E:T of 8:1. Shown is one representative experiment of two performed.*p < 0.05 **p < 0.02 by a two tailed student T-TEST.

The significance of ADAM17 in various aspects of NK cells activation was previously shown using short-term specific inhibitors [13, 17]. However, it is still unknown, if and how ADAM17 deficiency might affect NK cell function. To test whether the ADAM17 deficiency affects the expression of various NK cell receptors we stained the NK cells of the patient and of two healthy donors for the expression of central activating receptors and observed no significant differences (Figure 3A). To test the functionality of the various receptors, each of these receptors was tested in a redirected killing assay. We used P815 target cells, expressing an Fc receptor, and incubated them with various mAbs specific for a given activating NK receptor, thus activating each of these receptors individually. The NK cell receptors of the patient exhibited similar activity compared to healthy donors' NK cells, with only one exception, CD16, in which increased killing was noticed by the NK cells of the patient (Figure 3B).

We also performed direct killing assay using the K562 cell line which lysis is mediated by NKG2D and NCRs [18]. We observed no significant differences between the killing ability of the patient's NK and two healthy controls (Figure 3C). It was shown that CD16 is involved in the killing of 1106mel cells [19] [18]. We thus tested the killing of this cell line as well and in agreement with previous results [19], the patient NK cells kill better the 1106mel cells compared to the two healthy controls (Figure 3D).

**Figure 4:**
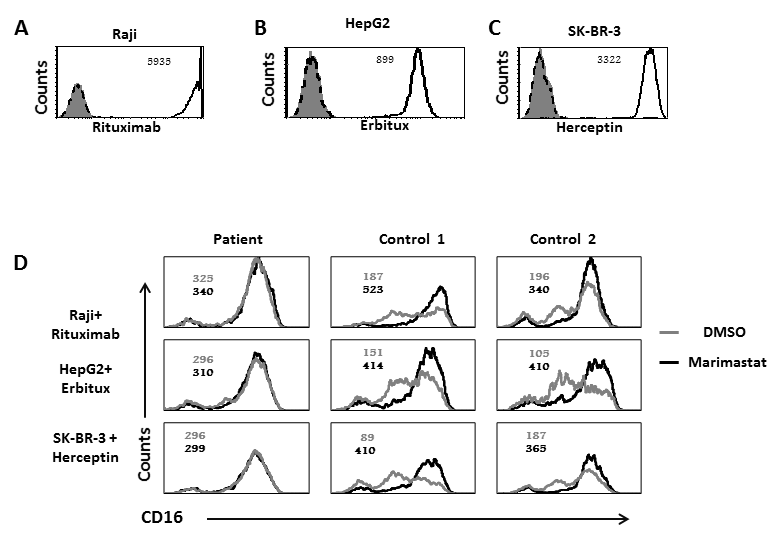
Reduced CD16 cleavage from the patients NK cells following CD16 stimulation by mAb **A.**-**C.** FACS analysis of Raji **A.** HepG2 **B.** and SK-BR-3 **C.** cells stained with Rituximab, Erbitux and Herceptin, respectively (empty black histogram), isotype control mAb (dashed black histogram) or secondary anti-human mAb (filled grey histogram). The MFI is indicated in the figure. **D.** Raji cells were coated with Rituximab (top), HepG2 cells with Erbitux (middle) and SK-BR-3 with Herceptin (lower). All targets were then incubated with IL-2 activated NK cells, derived from the ADAM17 patient or from two healthy controls treated either with DMSO (grey histograms) or with Marimastat (black histograms) at E:T of 4:1, for 5 hours. The NK cells were then stained with anti-CD16 mAb. The MFI of untreated NK is indicated in grey and the MFI of Marimastat treated NK is indicated in black. Shown is one representative experiment out of three performed.

Since we observed that ADAM17 deficiency affect CD16 activity we next analyzed the expression levels of CD16 following interaction with antibodies-coated cells. For these experiments we used Raji cells that express CD20 which is recognized by Rituximab (Figure 4A), HepG-2 cells that express EGFR, recognized by Erbitux (Figure 4B) and SK-BR-3 cells that express Her2, recognized by Herceptin (Figure 4C). We pre-treated NK cells from the patient and two healthy donors with DMSO (as control) or Marimastat, a known inhibitor of ADAM17 activity [20, 21], (emulsified in DMSO). Then we co-incubated the NK cells with Raji, HepG2, and SK-BR-3 cells coated with the appropriate mAbs: Rituximab, Erbitux or Herceptin, respectively. Following these co-incubations we observed significant cleavage (60%) of CD16 from the surface of NK cells obtained from the healthy donors, but not from the patient's NK cells (Figure 4D).

**Figure 5:**
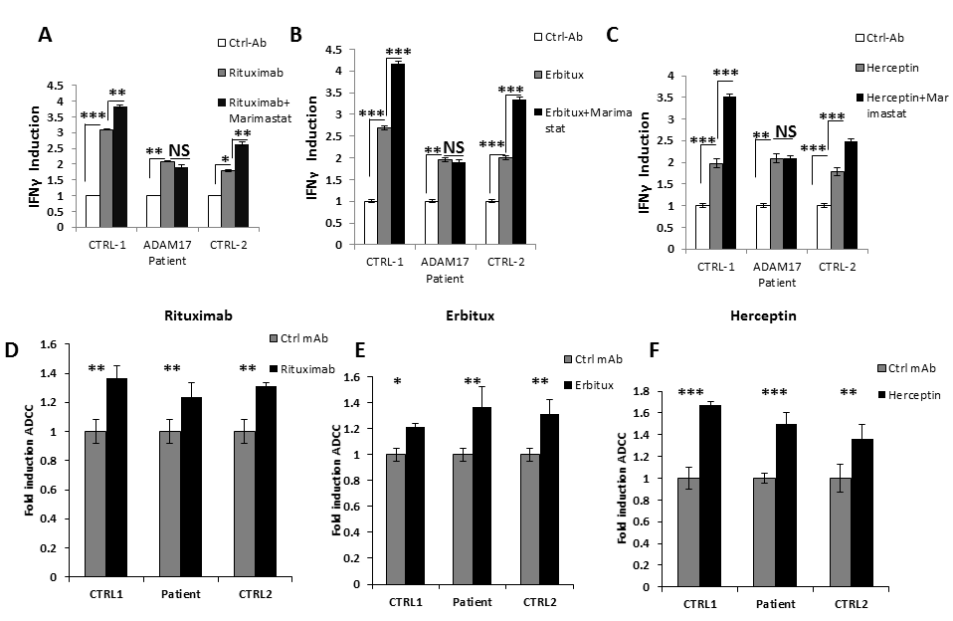
ADCC activity of ADAM17-deficient patients' NK **A.**-**C.** NK cells from two healthy donors (CTRL-1 and CTRL-2) and ADAM17 deficient patient (ADAM17) were pre-treated by DMSO or Marimastat and co-incubated with Raji **A.**, HepG2 **B.** and SK-BR-3 **C.**. Target cells that were coated or not with the indicated mAbs. IFNγ levels were measured following four hours co-incubation. Values are normalized to the control mAb secretion. *p < 0.03, **p < 0.003, ***p < 0.0005. **D.**-**F.**^35^S labeled Raji cells **D.**, HepG2 cells **E.**, or SK-BR-3 cells **F.** were incubated with the appropriate mAbs and then incubated with IL-2 activated bulk NK cell cultures derived from the ADAM17 patient and from two healthy control donors as indicated at the X axis, The effector to target ratios was 20:1. Shown is one representative experiment out of two performed. *p < 0.05, **p < 0.02, *** < 0.009 by a two tailed student *T*-TEST.

We next tested whether the ADAM17 deficiency will affect the antibody-related activities of CD16. For this purpose, we used Raji, HepG2, and SK-BR-3 cells coated with control mAb, Rituximab, Erbitux or Herceptin, respectively, and incubated them with NK cells from the patient and two healthy donors that were pre-treated with DMSO or with Marimastat. We hypothesized that the patients NK will not respond to Mariamstat treatment since ADAM17 does not exist in the patient. Incubation of all NK cells (derived either from the healthy controls or from the patient) with the specific mAbs induced IFNγ secretion (Figure 5A–5C). However, while Marimastat treatment led to a further secretion of IFNγ by healthy donors' NK cells, no further induction was observed by the patient's NK cells (Figure 5A–5C).

**Figure 6:**
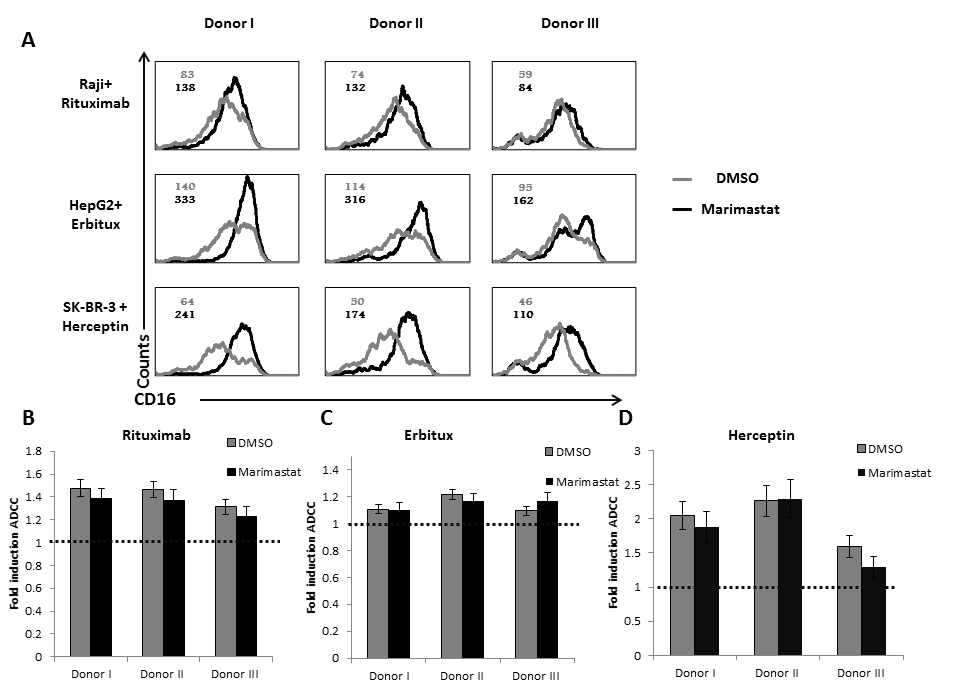
ADCC activity following ADAM17 inhibition **A.** Raji cells were coated with Rituximab (top), HepG2 cells with Erbitux (middle) and SK-BR-3 with Herceptin (lower). All targets were then incubated with IL-2 activated NK cells, derived three healthy donors, treated either with DMSO (grey histograms) or with Marimastat (black histograms) at E:T ratio of 8:1, for 5 hours. The NK cells were then stained with anti-CD16 mAb. MFI of untreated NK is indicated in grey and the MFI of Marimastat treated NK is indicated in black. **B.**-**D.** ^35^S labeled Raji cells **B.**, HepG2 cells **C.**, or SK-BR-3 cells **D.** were incubated with the appropriate mAbs and then incubated with IL-2 activated bulk NK cell cultures derived from the three healthy donors as indicated at the X axis, The effector to target ratios was 8:1. The basal killing activity was set as 1 (indicated by the dashed line), shown is the fold induction of ADCC by the specific mAb. Shown is one representative experiment out of two performed.

We next tested ADCC activity of the NK cells against Raji (Figure 5D), HepG2 (Figure 5E) and SK-BR-3 (Figure 5F) cells incubated either with control mAb or with the relevant mAbs. The addition of the appropriate mAb led to significant increase of killing of all targets tested. Surprisingly, no difference in the ADCC activity was observed between NK cell from the healthy donors and the NK cells of the ADAM17 patient although the CD16 levels of patient NK cells are not downregulated following mAb stimulation (Figure 4D). Thus, it seems that for ADCC a certain threshold of CD16 levels is sufficient for triggering of maximal ADCC. To test this hypothesis we treated NK cells from three different donors with DMSO or Marimastat. We next incubated the NK cells with Raji, HepG2, and SK-BR-3-coated with the appropriate mAbs: Rituximab, Erbitux or Herceptin, respectively. Following these co-incubations we observed significant cleavage (40-60%) of CD16 from the surface of NK cells treated by DMSO compared to the NK treated by Marimastat (Figure 6A). However, in spite of these differences, no increase at the induction of ADCC was observed when comparing the Marimastat and the DMSO treated NK cells (Figure 6B–6D).

## DISCUSSION

Boosting immune activity against tumor cells has gained much attention in the last years [22]. Several antibodies that are directed against tumor ligands are injected into patients and because one mechanism by which these anti-tumor mAbs operates is *via* ADCC, there is growing interest in boosting ADCC activity. This can be done by blocking the activity of ADAM17 (that cleave CD16) with chemical compounds [13] or by using specific antibodies [23].

So far, the function of ADAM17 *in vivo* was studied by using mice models in which ADAM17 deletion is embryonic lethal, and *in vitro* by using NK cells derived from healthy donors. Thus, it is still largely unknown what would be the outcome of prolonged treatment with anti-ADAM17 blocking compounds.

Only one previous report identified a deletion of ADAM17 in human patients [15]. The immunological status of the previous patients and ours are quite similar. However, in the report by Blaydon et al. IL-6 and IL-1b were secreted at similar levels while in our patient the secretion of both cytokines was impaired. This might be due the age differences between the patients or other genetic factors. Importantly, in the previous report and in our hands TNFα was barely secreted by the patient and in both cases this was accompanied by severe bacteremia.

Here we analyzed, for the first time, the NK cell phenotype and function in absence of ADAM17. It is important to emphasize that most of the results were obtained using IL-2 activated NK cells, a protocol that requires 10 days of in-vitro expansion and activation. We demonstrate that the patient and healthy donors express comparable levels of activating receptors. However, we showed that the CD16 activity on the patient's NK cells is enhanced. It had a better activity when it was cross-linked, and the NK cells of the patient killed better the 1106mel cell line (which is CD16-dependent). We further found that the patient's NK cells secrete IFNγ in comparable levels to the healthy controls, however, the treatment by Marimastat (a short-term inhibitor of ADAM17), boosted the IFNγ secretion by the NK cells from the healthy donors and not from the patient's NK cells. Overall, these findings suggest that activated NK cells were not affected by the recurrent infections of the patient and most of the differences observed might be related to the absence of ADAM17.

We observed no difference in ADCC activity of the patient as compared to healthy donors. Furthermore, when we compared ADCC activity of NK cells from healthy donors with or without Marimastat treatment there was no increase in the ADCC. These results were surprising as CD16 levels were significantly higher on NK cells during ADCC assays following Marimastat treatment. We suggest that for ADCC a certain threshold of CD16 levels is enough to induce maximal ADCC. Indeed, similar findings were reported by Romee et al. who showed that while IFNγ production and de-granulation were higher following ADAM17 inhibition, no increase in ADCC was observed [13].

Collectively, our results suggest that the short-term benefit of ADAM17 inhibition might lead to enhanced secretion of IFNγ, while long-term inhibition of ADAM17 might be detrimental and could increase the risk of bacterial infection due to drastic decrease in soluble TNFα.

## MATERIALS AND METHODS

### The following primers were used for genotyping

Forward: 5′- GCTGTGGAACTGGAGGGTAA-3′

Reverse 5′- TCAGCTCACTCACTGCAACC-3′

### Cytokine and viability tests

Briefly, 200,000 PBMC cells (ADAM 17 patient and two healthy controls) where cultured O.N in 96 well plates. Media was next changed to include 10ng or100ng LPS and incubated for additional night before collection. The treated media were assayed using Human Magnetic Luminex screen (R&D systems) measuring TNFα, IL6, IL6-rα, IL1β, IL1-Rα, IL10 and TNFRII according to manufacturer's instructions. Cell viability was tested with CellTiter-Glo (Promega)

### NK cell isolation

NK cells were isolated and activated as previously described [24]. Briefly, PBMCs derived from the patient or healthy donors by Lymphoprep™ density gradient (STEMCELL™ Technologies). NK cells were isolated by StemSep™ Human NK Cell Enrichment Kit. Following isolation, NK cells were incubated with; 6000RAD irradiated 5*10^6^ PBMCs from two donors and 5*10^5^ RPMI-8866 cells in presence of 400u/ml recombinant human IL-2 (PeproTech 200-02).

### Antibodies

The following antibodies were used: CD56-APC, NKG2D-PE, CD16-FITC(3G8), CD16 -PE B73.1, CD16-purified (3G8), NKp30-APC, NKp-46 PE, 2B4-APC, IFNγ-purified, IFNγ-biotin, anti-mouse biotin, anti-human APC, strepavidin-HRP. ADAM17 (ABT94) Merck Millipore, Rituximab, Herceptin, Erbitux.

### Cell lines

P815 mouse mastocytoma (ATCC^®^ TIB-64^™^), SK-BR-3 human adenocarcinoma (ATCC^®^ HTB-30^™^), Raji human Burkitt's lymphoma (ATCC^®^ CCL-86^™^), HepG2 human hepatocellular carcinoma (ATCC^®^ HB-8065^™^) cell lines were obtained from ATCC and maintained according to ATCC instructions.

### FACS staining

For all experiments with NK cells 10^5^ NK cells were used per well and the stainings were performed in 100μl volume using conjugated mAbs. For each fluorophore relevant isotype control was used. For staining of target cells final concentration of 10 μg/ml of the primary antibody was used in 100 μl volume.

### ^35^S-Met release assays,redirected killing assays and ADCC

The target cells were labeled with [^35^S]-Methionine 12 hours prior to the assay. Labeled targets (5000 cells/well) were incubated with different effector NK cells (various E:T ratios) as indicated in the figures. The assays were performed in RPMI medium in 96-U shaped plates at 37°C for 5 hours. Following incubation, plates were centrifuged (1600rpm, 5min, 4°C) and supernatants (50μl) were collected and transferred to opaque Opti-plates (Packard). 150μl scintillation liquid (Perkin Elmer) was added and analyzed by a micro beta, β-counter (Perkin Elmer). The maximal labeling was determined by adding 100μl of 0.1N NaOH to an equal amount of targets (5000/well). Spontaneous release was determined in wells containing target cells only. Final specific lysis was calculated as follows: ((radioactive reading - spontaneous release)/(maximal labeling - spontaneous release))*100 = specific lysis. In redirected assays P815 cells were incubated on ice for 1 hour with the indicated mAb at final concentration of 0.2 μg/ml prior to addition of NK cells. The killing of P815 cells in presence of control mIgG was subtracted from all specific mAbs values. In ADCC assays the targets were incubated with the relevant mAb/control mAb on ice for 1 hour at final concentration of 10 μg/ml prior to addition of NK cells. The killing of the targets in presence of control mAb was set as 1 and the induction of ADCC was calculated as follows (specific killing in presence of specific mAb)/(specific killing in presence of control mAb).

### Marimastat treatment

Marimastat or DMSO were added at 10μl/ml medium to NK cells for 1 hour. After 2 rounds of wash, NK cells were re-suspended in RPMI and used in the assay. In all ELISA assays, the the supernatants were taken for ELISA while the cells were used in FACS staining for CD16.

## SUPPLEMENTARY MATERIAL TABLE


