# Supplementary material for: Cytokine secretion and NK cell activity in human ADAM17 deficiency
Source: Oncotarget. 2015 Dec 16;6(42):44151–60. doi: 10.18632/oncotarget.6629 (PMC4792548; doi:10.18632/oncotarget.6629)
Supplement: Supplementary file 1 [file oncotarget-06-44151-s001.pdf]

Cytokine secretion and NK cell activity in human ADAM17 deficiency

Supplementary Material

Detailed clinical manifestation

The patient developed an erythematous eruption which was at times papulo-squamous and at times eczematous. The eruption was mainly peri-orifical and acral in distribution, but during exacerbations it became widespread. Alopecia of the scalp was noted, as well as sparse eyebrows, and eyelashes were noted. Beginning at one month of age he suffered from recurrent infections, including subcutaneous infections and bacteremias caused by *Staphylococcus aureus* and *Pseudomonas aeurginosa*, as well as perianal abscess due to *Enterobacter cloacae* and *Pseudomonas aeurginosa*, urinary tract infection due to *Pseudomonas aeruginosa*, recurrent pneumonia, purulent otitis externa due to *Staphylococcus aureus* , *Pseudomonas aeurginosa, Streptococcus pyogenes,* and *E. coli* and conjunctivitis due to *Staphylococcus aureus and Pseudomonas aeurginosa*. Echocardiography showed a large type II atrial septal defect with moderate pulmonary hypertension. He experienced recurrent bouts of severe watery diarrhea with hyper-osmolar dehydration. Endoscopy revealed mild inflammation of the duodenal lamina propria, without villous atrophy. Despite naso-gastric caloric supplementation he failed to thrive. At 19 months of age, he was hospitalized for pneumonia complicated by respiratory distress syndrome, resulting in chronic lung disease requiring mechanical ventilation. During a prolonged intensive care hospitalization he developed flaccid paralysis. MRI disclosed para-medial hyper-intense signal consistent with myelomalacia. An intra-abdominal cystic mass was noted. Attempts to aspirate the lesion percutaneously

were unsuccessful. The patient died at two years of age of respiratory failure and presumed sepsis. The parents declined postmortem examination. Studies were performed with informed consent according to a protocol approved by the Hadassah Ethics Committee

Supp. Table1:

	Untreated		LPS 10ng		LPS 100ng	
	P-value Patient vs Con-1	P-value Patient vs Con-2	P-value Patient vs Con-1	P-value Patient vs Con-2	P-value Patient vs Con-1	P-value Patient vs Con-2
TNFa	0.023488902	0.006397342	0.013753401	0.025233603	0.000665139	0.001317163
IL6	0.019010411	0.001851349	0.00890546	0.009391593	0.002337312	0.000440891
IL6-ra	ND	ND	ND	ND	ND	ND
IL1-b	0.052080845	0.00422688	0.000389	0.059769373	0.002788461	0.000126935
IL1-Ra	0.0385228	0.01848416	0.031822677	0.019106442	0.001493333	0.000311761
IL10	ND	ND	0.008163402	0.057190958	0.008163402	0.026271009
TNFRII	0.018844219	0.00747674	0.013072458	0.019803941	0.053270738	0.063414188
